# Comparative Epidemiological Investigation of Alzheimer’s Disease and Colorectal Cancer: The Possible Role of Gastrointestinal Conditions in the Pathogenesis of AD

**DOI:** 10.3389/fnagi.2018.00176

**Published:** 2018-09-28

**Authors:** Tianlang Zhang, YaRu Han, JianYi Wang, Deren Hou, Hao Deng, Yun L. Deng, Zhi Song

**Affiliations:** Department of Neurology, Third Xiangya Hospital of Central South University, Changsha, China

**Keywords:** Alzheimer, colorectal cancer, constipation, gut-brain axis, gut microbiota alteration

## Abstract

Alzheimer’s disease (AD) is a neurodegenerative disorder that affects approximately 35 million people worldwide, and diet has been reported to influence the prevalence/incidence of AD. Colorectal cancer is among the most common cancers in Western populations, and the correlation between constipation and the occurrence of colorectal cancer has been identified in a number of studies, which show that a Westernized diet is a mutual risk factor. Constipation is a growing health problem, particularly in middle-aged and older adults. As the most common gastrointestinal disorder in adults, constipation affects 2–20% of the world population, and it is associated with several diseases, such as diabetes, Parkinson’s disease, and others. Comparing the epidemiological data on colorectal cancer and AD, we find that colorectal cancer and AD have similar epidemiologic feature, which is both disease correlate with high prevalence of constipation. Therefore, we hypothesized that constipation may influence Alzheimer’s disease in a similar way that it contributes to colorectal cancer. This review aimed to systemically elucidate the evidence that constipation contributes to Alzheimer’s disease progression.

## Introduction

Alzheimer’s disease (AD) is a chronic neurodegenerative disease which was first described by Alois Alzheimer (Grant, 2016). The disease usually has concealed onset and gets progressively worse. It is characterized by irreversible loss of memory and cognitive decline. It is generally considered the most common dementia subtype. To date, it is estimated that approximately 42 million people now have dementia globally. Aging is a crucial risk factor for AD, with the aging of the population becoming increasingly more common, AD is becoming one of the most serious threats to public health. So far, the cause of the disease is still debated, and environmental and genetic factors seem to be critical influences on AD.

In recent years, the relationship between diet patterns and AD has drawn the attention of researchers. According to the worldwide epidemiological investigation of Alzheimer’s disease, the Europeans and Americans are believed to have higher prevalence and incidence rates of AD. According to worldwide diet pattern research, there are some differences between the Eastern and Western diets. Westernized diets tend to include meat, sweets, and high-fat dairy products, which are found to be risk factors for AD (Grant, 1997); for instance, when Japan made a nutritional transition from the traditional Japanese diet to a Western-pattern diet, AD rates rose from 1% in 1985 to 7% in 2008 (Dodge et al., 2012). That is, the Western diet may be a risk factor for Alzheimer’s disease.

Additionally, diet is one of the factors that influence constipation. Through global epidemiological investigations, we found that, compared with North America, Europe and Oceania, Asian countries seem to have lower constipation morbidity rates (Mugie et al., 2011).

In addition, when Japanese people transformed their diet pattern from a traditional diet to a Western diet, not only did AD incidence increase dramatically but also their risk of colon cancer was much higher (Tominaga and Kuroishi, 1997).

Colorectal cancer, with higher incidence in Western countries and lower incidence in Eastern countries, has been found in many studies to be related to high meat consumption and low vegetable intake, which is the major food pattern of Westernized diets. This pattern is one of the main causes leading to high colorectal cancer incidence (Boyle and Langman, 2000), and a correlation between constipation and colorectal cancer has been found in the majority of studies.

Therefore, in this essay, we aim to present the possible epidemiological resemblance between constipation and Alzheimer disease, and put forward the hypothesis that diet and constipation might impact the progression of Alzheimer’s disease in a similar way that they influence colorectal cancer.

## The Association Between Epidemiological Investigation of Alzheimer’s Disease, Colorectal Cancer and Constipation

### Epidemiological Investigation of AD

In 2015, there were approximately 29.8 million people worldwide suffering with Alzheimer’s disease. Alzheimer’s disease is well-known as the main cause of dementia and generally considered to produce low later life quality (Vos et al., 2016). Alzheimer’s disease is characterized by memory loss, extracellular amyloid plaque deposition involving the Aβ peptide and intracellular tangles of tau protein accompanied with reactive microgliosis, dystrophic neurites, and loss of neurons and synapses (Konietzko, 2015), and alteration in the production and processing of amyloid β-protein has been hypothesized as the major initiating factor. However, the underlying causes of these pathological changes remain unclear, but increasing age and genetic and non-genetic factors may be essential influences (Serrano-Pozo et al., 2011).

The prevalence of AD has been found to be associated with increasing age; for instance, as age increases from 65–74 years to 85 years or older, the prevalence of AD, correspondingly, rises from 3 to 50% (Castellani et al., 2010). With the worldwide incidence rate of AD increasing rapidly, AD is becoming one of the most serious threats to public health. In the United States, the prevalence of AD is estimated to experience double-digit to triple-digit percentage increases between 2010 and 2025 (Weuve et al., 2015).

According to several worldwide epidemiological investigations of Alzheimer’s disease, we found that Europeans and Americans are believed to have higher prevalence and incidence rates of AD. Some researchers identified 27 studies reporting age-specific incidence rates for AD, from which they found that the incidence of AD at age 80 in Europe and North America was higher than that in other countries (Ziegler-Graham et al., 2008). Lobo et al. (2000) compared the incidence data from eight population-based studies in Europe, which suggested that prevalence of AD in those >65 years of age was 4.4%, which was higher than that of Nigeria (1.4%) and India (1.1%) (Lobo et al., 2000). Moreover, a meta-analysis of the prevalence and incidence of dementia due to Alzheimer’s disease provided more specific data; within the community settings (30.4 per 1000 persons), the estimated annual period prevalence for North America was 103.6 per 1000 persons, which was much higher than the annual period prevalence of Asia (11.7 per 1000 persons) and that in Europe (31.3 per 1000 persons) (Fiest et al., 2016).

In 2005, Alzheimer’s Disease International managed an evidence-based Delphi consensus on dementia prevalence worldwide, and the Delphi study showed that in 2001, an estimated 24 million people had dementia (Ferri et al., 2005); the majority of these people are thought to have had Alzheimer’s disease. There is one research study that provided dementia data on people aged above 60 years in different places, and the data indicated that the prevalence in North America was 6.3%, Western Europe was 5.3%, Latin American was 4.9%, China was 4.0%, and the Western Pacific area was 4.0% (Ferri et al., 2005); people from North America and Western Europe had the highest incidence, and China and the Western Pacific area had the lowest incidence. Moreover, similar conclusions were drawn in several passages in a 2012 Alzheimer’s disease epidemiology paper in which the author found that dementia prevalence using DSM-IV criteria was extremely low in low-income places, and the author declared that dementia prevalence was estimated to be 4.7% worldwide, with Americans at 6.5%, Europeans at 6.2%, and Asians at 3.9% (Sosa-Ortiz et al., 2012).

In particular, Japan is a Westernized Asian country, and compared to other Asian countries, Japan has relatively higher prevalence/incidence of some diseases. One study described the trends in Alzheimer’s disease prevalence in Japan as 1.5% in 1985, 1.4% in 1992, 2.4% in 1998, 3.9% in 2005, and 7.2% in 2012 (*p* for trend <0.01); the increasing trend is apparent, and we chose the most recent AD prevalence data from Japan for the figure for better comparison (Ohara et al., 2017).

Based on the data we collected from diverse research, we present our information in **Table 1**.

**Table 1:** Characteristics of included studies.

Region	Country	Study	Time	Dementia prevalence
North America		Prince and Jackson, 2014	2009	6.46
	United States	Rocca et al., 2011	2001	7.5
Latin America		Mayeux and Stern, 2012	2012	4.9
	Brazil	(1) Scazufca et al., 2008	2002–2008	5.3
		(2) Cerqueira et al., 2005		
		(3) Bottino, 2007		
		(4) Herrera et al., 2002.		
	Argentina	Pages-Larraya and Mari, 1999	1999	11.5
	Colombia	(1) Rosselli et al., 2000	2000	2.1
		(2) Pradilla et al., 2002		
Europe		Sosa-Ortiz et al., 2012	2012	6.2
Western Europe		Mayeux and Stern, 2012	2012	5.3
	Greece	Tsolaki et al., 1999	1993	9.59
	Spain	Lobo et al., 2000	1990–2008	9.8
	France	Obadia et al., 1997	1997	9.2
	Sweden	Qiu et al., 2013	1990–2000	17.7
	Norway	Engedal and Haugen, 2004	1880–1920	16.3
	England	Matthews et al., 2013	2011	8.3
	Finland	Sulkava et al., 1985	1985	9.2
	Italy	Rocca et al., 1990	1990	6.2
	Holland	Breteler et al., 1992	1992	8.5
Asia		Sosa-Ortiz et al., 2012	2012	3.9
Southeast Asia		Prince and Jackson, 2014	2009	6.38
Western Pacific		Mayeux and Stern, 2012	2012	4.0
	India	Sosa-Ortiz et al., 2012	2012	1.1 (AD)
	South Korea	Kim et al., 2011	2008	9.2
	Japan	Ohara et al., 2017	2012	7.2
	Taiwan, China	(1) Lobo et al., 2000	1995–1998	3.2
		(2) Liu et al., 1998		
		(3) Liu et al., 2015		
	China mainland	Dong et al., 2007	1980–2004	3.1
	Turkey	Arslantas et al., 2009	2009	8.4
Other countries				
	Nigeria	Sosa-Ortiz et al., 2012	2012	1.4 (AD)
	Australia	Prince and Jackson, 2014	2009	6.91


From these studies, we can infer that the prevalence of dementia and Alzheimer’s disease was lower in developing or low and middle income countries [11, 12, 16, 17]; the difference between the developed and developing countries was very large in some studies (Hendrie et al., 1995; Chandra et al., 1998). However, there were a number of statistics about the studies on Alzheimer’s disease and dementia prevalence; Western Europe had 61 studies, and East Asia had 34 studies. The high income area of Pacific Asia had 22 studies, North America had 13 studies, Latin America had 11 studies, South Asia had 7 studies, South East Asia had 5 studies, Australasia had 4 studies, and there were also some places with just a few studies. The Caribbean had 4 studies, Central Europe had 4 studies, North Africa/Middle East had 2 studies, Eastern European had 1 study, and Southern sub-Saharan Africa had 1 study; even though we have known that since the mid-1990s the number of studies in low income countries has increased dramatically, it is clear that there are still more studies in developed countries, and to assume that developing countries have lower incidence, we need more valid studies in those countries.

### Epidemiological Investigation of Colorectal Cancer

Colorectal cancer is one of the most common cancers in Western countries, and its incidence/prevalence has tended to increase worldwide. At the end of the 20th century, approximately one million colorectal cancer cases came up worldwide every year, almost equal to one tenth of all new cases of cancer (Stewart and Kleihues, 2003).

In the United States, colorectal cancer is the third most common cancer and the second most common cause of cancer death. Among all these countries worldwide, India has the lowest rates, and Japan has the highest rates. Moreover, researchers found that colorectal cancer incidence rates increase acutely with age, and colon cancer occurs equally in both sexes, whereas rectal cancer occurs twice as frequently in men as in women (Potter and Lindor, 2009). Colorectal cancer incidence rates vary dramatically all over the world, and as of 2012, it was the second most common cancer in women (9.2%) and the third most common cause of cancer in men (10.0%); it was the fourth most common cause of cancer death. In 2014, there were 71830 men and 65000 women estimated to be diagnosed with this disease, and more than a third of them will die from this disease (Siegel et al., 2014).

Western societies are thought to have higher colorectal cancer incidence rates (Boyle et al., 1985). However, studies from the Cancer Base of the International Agency for Research on Cancer (IARC) showed that the incidence rate in some developed and Westernized Asian countries, especially Japan, are now increasing to almost the same level as the West (Sung et al., 2005).

**FIGURE 1:**
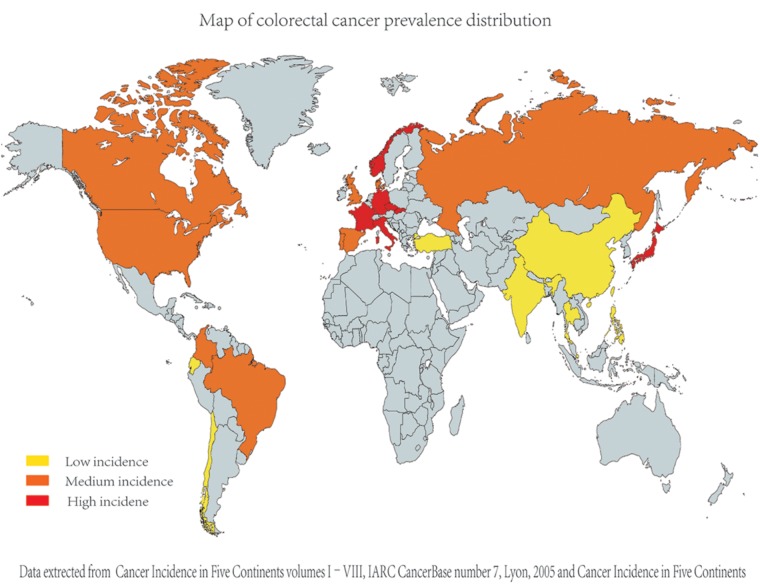
Comparison of different countries’ colorectal cancer incidence (age standardized incidence per 100000). As the epidemiological data on colorectal cancer were collected from diverse studies [including Cancer incidence in five continents (Curado et al., 2007)], their criteria and dates are apparently different; for better comparison, we evaluated and compared the data in each study and divided the data into three groups (relatively high, relatively medium, and relatively low group) artificially.

Based on the studies, we found that, generally, worldwide colorectal cancer prevalence is higher in Western countries and lower in Eastern countries. The collection of colorectal cancer epidemiology data is shown in **Figure 1** (Sung et al., 2005; Center et al., 2009).

According to the research of international variation and immigration data, colorectal cancer has been shown to be highly sensitive to environmental factors. One passage gave the example that immigrants and their offspring reach the local cancer morbidity number in a short time (Potter and Lindor, 2009). Diet and other local cultural factors are possibly major causes. Moreover, the majority of the case-control studies showed that meals with more vegetables and fruit are correlated with lower risk of colorectal cancer (Potter and Lindor, 2009). Many studies have shown that Eastern countries tend to have lower incidence of colorectal cancer than Western countries. According to studies about colorectal cancer epidemiology, diets high in fat, meat, animal protein and low in fiber, vegetables and fruits is an important risk factor (Boyle and Langman, 2000), which is also a risk factor for Alzheimer’s disease.

### Epidemiological Investigation of Constipation

Constipation is a very common gastrointestinal disease affecting all age group, and people tend to define it as bowel symptoms (difficult or infrequent passage of stool, stool hardness, or a feeling of unfinished evacuation). Based on the definition, with either self-reported or Rome criteria, constipation occurs in 2–20% of the population (Locke et al., 2000). It troubles patients physically and mentally and dramatically affects people’s daily life and well-being; additionally, the health costs of constipation are tremendous; in the United States, constipation-relevant health treatment costs 6.9 billion dollars every year, and constipation is reported to occur more frequently in the elderly; as life expectancy is increasing, an elevation in the prevalence of constipation is promised to come, alongside negative effects on life quality and the burden to society and the economy.

For a disease people are familiar with, constipation has actually quite high incidence in human kind, and to further study it, research from diverse countries have collected data. Pu-Lin et al. (2001) used a cluster random sampling method to investigate the elderly (≥60 years) in six cities (Beijing, Shanghai, Guangzhou, Xian, Shenyang, Chengdu) in China; they found that the crude prevalence rate of constipation is 11.5% (Pu-Lin et al., 2001); a constipation epidemiology study in the United States showed that the overall prevalence of constipation was 14.7% (Stewart et al., 1999); one study showed the pooled prevalence of chronic idiopathic constipation in diverse countries as follows: South East Asia was 11%, North America was 14%, Northern Europe was 16%, Southern Europe was 16%, and South America was 18% (Suares and Ford, 2011). One research study revealed that the prevalence of constipation in the general population (in Germany) was 14.5%, which was much higher than their previously reported prevalence and similar to data in other European countries (Enck et al., 2015). A survey according to Rome I criteria described the constipation prevalence in Canada to be 16.7% (Pare et al., 2001).

One study described that the Japanese prevalence of constipation in men was 11.9%, and it was 31.8% in women; overall, it was 23.0% (Watanabe et al., 2004).

The information of individual studies is shown in **Table 2**.

**Table 2:** Characteristics of included studies.

Region	Country	Study	Time	Constipation prevalence
North America		Suares and Ford, 2011	2011	14
	United States	Stewart et al., 1999	1999	14.7
	Canada	Pare et al., 2001	2011	16.7
South America		Suares and Ford, 2011	2011	18
	Brazil	Wald et al., 2008	2010	16.7
	Argentina	Wald et al., 2009	2010	14.2
	Colombia	Wald et al., 2009; countries	2010	21.7
Northern Europe		Suares and Ford, 2011	2011	16
Southern Europe		Suares and Ford, 2011	2011	16
	Germany	Enck et al., 2015	2015	14.5
	Greece	Papatheodoridis et al., 2010	2006	14
	France	Siproudhis et al., 2006	2003	22.4
	Sweden	Mjörnheim et al., 2003	2001	31.4
	Norway	Haug et al., 2002	2002	20.2
	England	Thompson and Heaton, 1980	1980	20.6
	Finland	Kinnunen, 1991	1991	29
	Italy	Gaburri et al., 1989	1989	9.2
	Holland	van Kerkhoven et al., 2008	Late 1980s- Early 1990s	14.2
	Spain	Garrigues et al., 2004	2004	29.5
South East Asia		Suares and Ford, 2011	2010	11
	Taiwan, China	Lu et al., 2006	2001	8.5
	China mainland	Pu-Lin et al., 2001	2001	11.3
Other countries				
	South Korea	Kyo et al., 2000	2000	24.3
	Japan	Watanabe et al., 2004	1990	23
	Australia	Chiarelli et al., 2000	2000	27.7
	Turkey	Pamuk et al., 2003	2003	29.8


### The Possible Relationship Between AD and Constipation

Eastern countries appear to have lower constipation and dementia prevalence through observing the Alzheimer’s disease and constipation prevalence data in **Tables 1**, **2**. Based on the similarity of the prevalence distributions of these two diseases, we speculate that Alzheimer’s disease may correlated with constipation, which means either they may share a common factor, or one disease could contribute to the occurrence of the other disease.

To demonstrate the relation between constipation and AD, data in **Tables 1**, **2** which did not meet criteria, including comparable study year and area, were excluded. Statistical analysis was used to analyze the correlation between AD and constipation. Dementia prevalence was used to do the correlation analysis as AD makes up almost 70% of dementia (Burns and Iliffe, 2009).

**FIGURE 2:**
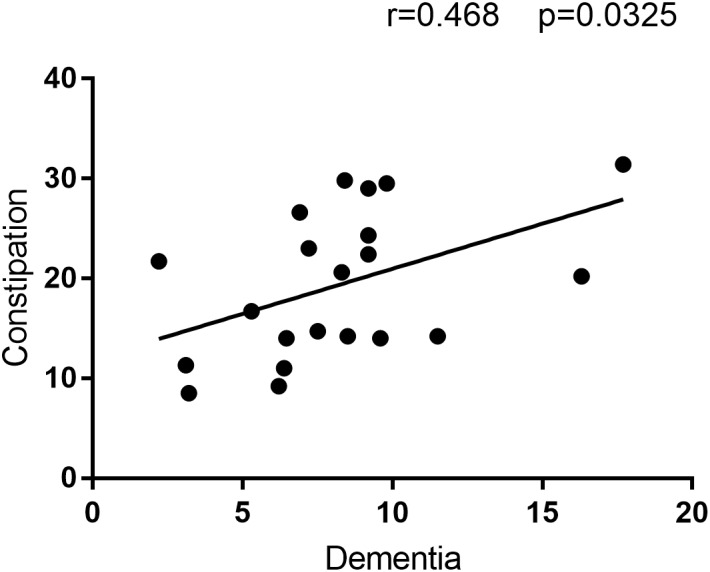
The correlation analysis result between Constipation and Dementia prevalence. This figure is the result of correlation analysis between constipation prevalence and Dementia prevalence. Constipation prevalence is the *Y*-axis, and Dementia prevalence is the *X*-axis. *R* = 0.468 means the correlation coefficient of this analysis is 0.468, and alongside with *P* = 0.0325 < 0.05 indicate constipation prevalence is positive correlated with Dementia prevalence.

The correlation analysis revealed a positive correlation between Dementia prevalence and constipation prevalence (**Figure 2**). Hence, the location with higher constipation prevalence inclined to have higher Alzheimer’s disease prevalence, which may further prove the hypothesis that these two disease are related or one disease is involved in the pathogenesis of the other disease.

## A Possible Mechanism Underlying the Association Between Constipation and AD

### Gut-Brain Axis and Gut Microbiota

The gut and the brain are connected through the gut-brain axis (Rhee et al., 2009). The CNS communicates with the gut nervous system, enteric mucosa and muscle layer via bidirectional (i.e., afferent and efferent) paths and can control enteric movement, immune reaction and mucus secretion. The central nervous system, autonomic nervous system, brain and spinal cord, enteric nervous system, and the hypothalamic pituitary adrenal are part of this bidirectional pathway (Carabotti et al., 2015).

The gut-brain axis can connect brain emotional and cognitive centers with intestinal function, and also, as one significant part of the intestinal environment, gut microbiota have been shown to be important in the gut and brain interaction. It was suggested by clinical data and studies that gut microbiota could locally influence the intestinal cells and enteric system. Moreover, gut microbiota can directly interact with the central nervous system by neuroendocrine and metabolic paths(Carabotti et al., 2015). The role of microbiota in the gut-brain axis was first discovered more than 20 years ago when patients with hepatic encephalopathy improved their condition dramatically with administration of oral antibiotics (Morgan, 1991). Meanwhile, increasing studies showed that the gut microbiota are one essential part influencing anxiety and depression (Foster and Neufeld, 2013; Naseribafrouei et al., 2014). Moreover, the association between gut bacteria alteration and the severity of symptoms has been observed in autism patients (Song et al., 2004; Mayer et al., 2014).

Gut-brain axis disruption could cause gastrointestinal motility and secretion alteration and lead to visceral hypersensitivity, giving rise to cellular transformation of the immune system and gastrointestinal endocrine system. Gut microbiota may play an essential role in those diverse pathophysiological mechanisms. Crouzet L and colleagues showed that visceral sensitivity, which is characterized by irritable bowel disease, could be shifted to germ-free rats by using the gut bacteria of irritable bowel disease patients.

Research on germ-free animals has revealed that the gut microbiota is essential in central nervous system and enteric nervous system development and maturation (Barbara et al., 2005; Stilling et al., 2014).

Evidence shows that the gut microbiota interacts with the brain through the vagus nerve, which could connect the luminal environment with the central nervous system. Gut microbiota may also interact with the gut-brain axis by affecting the sensory nerves. There have been reports that *Lactobacillus reuteri* could increase the excitability of the nerves by suppressing the calcium-dependent potassium channels; thus, gut microbiota would be able to influence gut motility and pain perception (Kunze et al., 2009). By generating substances that can function local transmitters, such as GABA, serotonin, histamine, melatonin, and acetylcholine (Iyer et al., 2004), and creating active state catecholamines in the gastrointestinal lumen, gut microbiota can affect enteric nervous system excitability (Asano et al., 2012). Moreover, gut microbiota have shown the ability to activate the gastrointestinal mucosal immune system (Verdu et al., 2006).

One principle product of gastrointestinal bacteria is short-chain fatty acids, which include butyric acid, propionic acid, and acetic acid, and the enteric nervous system can be affected by these substances; these metabolites can also influence the sympathetic system (Kimura et al., 2011), increase mucosal serotonin release and affect learning and memory function (Vecsey et al., 2007; Stefanko et al., 2009).

On the other hand, the brain can also affect the gut microbiota. It has also been shown that under the influence of short stressors, the gastrointestinal microbiota can change dramatically. After only a 2-h social stressor, the bacteria community alteration and the reduction of main microbiota phyla is evident. In addition, the brain can influence gastrointestinal bacteria function and composition by modulating intestinal permeability, which leads to the penetration of gastrointestinal bacteria through the gut epithelium, thus producing an immune reaction in the gastrointestinal mucosa (Santos et al., 1998). Input from the gastrointestinal system to the CNS can also contribute to several symptoms, and the CNS and gastrointestinal system are linked via the gut-brain axis.

### Constipation and Gastrointestinal Condition Alteration

Constipation is a worldwide disease that affects almost one-third of the general population during their lifetime; its chronic symptoms seriously impair patient life quality and produce heavy economic burden for patients and society (Sun et al., 2011; Guerin et al., 2014).

The gut microbiota have an important role in maintaining gastrointestinal environment stability. It is reported that gut microbiota are correlated with gastrointestinal motility (Barbara et al., 2005), and another group demonstrated that intestinal bacteria could influence the migrating myoelectric complex, which are waves of electrical activity that sweep through the intestine in a regular cycle during fasting in a germ-free rat model (Husebye et al., 2001). Some studies showed that gut microbiota could affect gut immune response system to release mediators, which are bacterial fermentation end-products and intestinal neuroendocrine factors, to influence gastrointestinal motor ability (Barbara et al., 2005; Quigley, 2011a; Wu et al., 2013). Additionally, gastrointestinal microflora play an essential role in the human body to provide nutrition, which regulates the gastrointestinal epithelium and modulates innate immunity.

The cause of constipation is still unclear, and the alteration of gut microbiota has been shown to be one possible pathophysiologic mechanism (Attaluri et al., 2010; Belsey et al., 2010; Quigley, 2011b). An experiment using humanized (ex-germ-free mice transplanted with human fecal microflora) mice described that the change in gastrointestinal motility could lead to the alteration of gut microbiota; furthermore, with the administration of polyethylene glycol or a non-fermentable cellulose-based diet, which affects gastrointestinal motility, the mice exhibited similar alteration of gut microbiota (Kashyap et al., 2013). Regardless of whether the gut microbiota composition variation is a cause or a consequence of constipation, the correlation between gut microbiota alteration and constipation was shown in this experiment.

Another study Khalif et al. (2005) demonstrated that relief of constipation tends to normalize the gut microbiota and concluded that gastrointestinal flora alteration is more likely to be the result of constipation rather than the cause of it.

By far, the abundance or lack of certain kinds of bacteria due to constipation is poorly characterized, and there are contradicting data. Kim et al. (2015) used quantitative real-time polymerase chain reaction to investigate the gut microbiota traits in patients with functional constipation. They found that *Bifidobacterium* and *Bacteroides* species had lower abundance in feces from the functionally constipated patients compared with the control group, and no significant differences were found in the proportion of *Lactobacillus*, *Escherichia coli* and *Clostridium species*. Another study found that *Bifidobacterium* and *Lactobacillus* were less abundant in adult patients with constipation (Khalif et al., 2005). However, a study of children with constipation revealed that the abundance of *Bifidobacteria* and *Clostridia* were increased in their feces compared with the control group (Zoppi et al., 1998). Another study found significantly decreased levels of *Prevotella* in patients with constipation by using 16S rRNA gene pyrosequencing (Zhu et al., 2014).

### Gut Microbiota Alteration and AD

The intestinal bacteria are defined as an aggregation of gut living microorganisms, and their concentration is approximately 10^11^–10^12^cells/g in the gastrointestinal cavity. The amount of gastrointestinal bacteria is almost 10 times than that of human body cells.

Gut microbiota play an important role in maintaining a normal gut environment and modulating the signal along the gut-brain axis. Gut microbiota alteration and the increase in intestinal permeability may cause an overall systemic inflammation, neuroinflammation and dysfunction of particular brain regions, such as the cerebellum and hippocampus (Daulatzai, 2014a,b), and may also cause insulin resistance, which has been shown to be related to AD pathogenesis (Bekkering et al., 2013; Alam et al., 2014; Naseer et al., 2014).

No certain evidence of gut microbiota alteration exists in AD patients yet. Nevertheless, gut microbiota alteration has been found in patients with multiple sclerosis and Parkinson’s disease, in which neuroinflammation and protein misfolding were observed. Moreover, Berer et al. (2011) reported that removing gut microbiota in the animal models of multiple sclerosis could hold back the progression of repalsing-remitting demyelination (Berer et al., 2011), and other studies described that oral ingestion of probiotics could relieve neuroinflammation (Luo et al., 2014; Toumi et al., 2014).

Cattaneo et al. (2017) investigated the relationship between gut microbiota alteration and brain amyloidosis. Participants in this study were divided into three groups: cognitively impaired patients with amyloidosis (Amy+ group), cognitively impaired patients without amyloidosis (Amy- group) and cognitively healthy patients without amyloidosis (control group); the stool bacteria abundance and blood inflammation biomarkers were measured and compared among those three groups. The results showed that the Amy+ group had a unique bacteria alteration pattern, which was a smaller amount of *E. rectale* and larger amount of *Escherichia/Shigella* in stool compared with the other two groups, and furthermore, compared to the control group, the Amy+ group had a lower abundance of *Bacteroides fragilis.* The Amy+ group also showed higher serum concentrations of four cytokines (NLRP3, CXCL2, IL-6, and IL-1β) compared to the Amy- group and the control group. This study demonstrated the correlation among cytokines IL-1β, NLRP3 blood concentration and *Escherichia/Shigella* in the Amy+ and Amy- group, and the absence of correlation between IL-1β and CXCL2 with *E. rectale* in the Amy+ and Amy- groups; thus, the study concluded that the increase in pro-inflammatory gut bacteria, e.g., *Escherichia/Shigella*, and the decrease in anti-inflammatory gastrointestinal bacteria, e.g., *E. rectale*, might be one part of Alzheimer’s disease pathology (Cattaneo et al., 2017).

Moreover, endotoxin, as a bacterial product, has been discovered within senile plaques of Alzheimer’s disease brain tissue (Hauss-Wegrzyniak et al., 2000). It has also been reported that the bacteria endotoxin is correlated with Alzheimer’s disease pathological processes and amyloidosis. One study investigated the possible causes of Aβ plaque formation and reported that *E. coli* endotoxin could potentiate Aβ fibril formation *in vitro*, which could be part of AD pathogenesis (Asti and Gioglio, 2014).

It has been shown that the presence of an undefined number of LPSs (lipopolysaccharides) and amyloid plaques in the human gastrointestinal tract might participate in neuronal pathogenesis characterized by amyloidogenic features, such as AD (Hammer et al., 2008; Marques et al., 2013; Tran and Greenwood-Van Meerveld, 2013; Hill and Lukiw, 2015; Shoemark and Allen, 2015). However, this hypothesis has not been tested.

During aging, the gut epithelium and blood-brain barrier tend to have higher permeability to small molecules. Therefore, LPSs, amyloids and other complexes excreted by fungi and bacteria in the gut more easily get through (Marques et al., 2013; Tran and Greenwood-Van Meerveld, 2013; Hill and Lukiw, 2015; Shoemark and Allen, 2015); thus, amyloid formation and dissemination caused by gut microbiota might be more influential in the elderly. Other studies have shown that the receptor for advanced glycosylation products (RAGE) and apolipoprotein E and J control the amyloid influx through the blood–brain barrier (Zlokovic, 1996; Rashid et al., 2003), while low density lipoprotein receptor-related protein monitors amyloid clearance (Deane et al., 2004), which could change in AD patients (Weiss et al., 2009).

To date, these research studies show evidence that gut microbiota alteration may participate in Aβ plaque formation.

The correlation between constipation and Alzheimer’s disease has been rarely mentioned before. However, there are some links between them. First, AD and constipation share a common risk factor, which is the high-fat-low-fiber diet pattern. Second, according to epidemiological data, the locations with high prevalence of Alzheimer’s disease also have relatively high constipation prevalence. Third, constipation is associated with gut microbiota alteration, which may contribute to the occurrence of Aβ plaque formation.

Therefore, after evaluating evidence of the link between AD and constipation, we proposed the hypothesis that constipation may contribute to the occurrence of AD, and improving constipation for patients before the onset of AD may interfere with AD progression.

## Summary and Perspectives

The prevalence/incidence data analysis showed that Asian region have lower constipation prevalence and lower Dementia prevalence compared to other regions, which indicate Alzheimer’s disease and constipation may be related or share a common factor. Moreover, the statistical positive correlation between AD and constipation lead us to think that constipation influenced Alzheimer’s disease as with it affected colorectal cancer.

Furthermore, evidence shows the possible association between constipation and Alzheimer’s disease. The gut-brain axis is a bidirectional path, and the gut microbiota play an important part in it. Through this bidirectional pathway, input from the gastrointestinal system to the CNS can contribute several symptoms, and the brain can affect gut microbiota function and composition. Gut microbiota participate in maintaining a normal gastrointestinal environment and have been shown to affect gastrointestinal motility. In return, a change in gut motility can also lead to gut microbiota alteration, and constipation has been thus demonstrated to be relevant to gastrointestinal bacteria alteration. Studies have demonstrated that the gut microbiota impact the neuronal system and the direct or indirect correlation between gut microbiota alteration and amyloidosis. For instance, the *E. coli* endotoxin has been shown to contribute to the formation of Aβ fibrils *in vitro*, and amyloids and LPSs have been found in the gastrointestinal tract, which might participate in brain amyloidosis. Furthermore, the blood-brain barrier and gastrointestinal epithelium tend to have higher permeability in the elderly, thus the consequences of gut microbiota alteration and toxic products of gastrointestinal bacteria may affect the brain more. Therefore, we propose the hypothesis that constipation may be related to Alzheimer’s disease pathological processes.

As modern medicine faces a lack of effective Alzheimer’s disease treatment, therapeutic approaches that could aid in preventing and/or delaying the onset of AD are required; thus, we present the idea that ameliorating constipation in patients before the onset of Alzheimer’s disease may prevent or delay Alzheimer’ disease progression.

## Study Limitation

This study has some limitations. First, criteria and time of data collection across studies were different, which might cause some inaccuracy in our research; however, there are several studies that show that AD prevalence is lower in developing countries or lower income areas (Hendrie et al., 1995; Chandra et al., 1998; Prince, 2000; Ferri et al., 2006); thus, AD incidence worldwide probably fits our assumption.

Second, population aging trends in developed countries might influence the AD epidemiology results. AD prevalence increases exponentially with age; for instance, among those who are 65–74 years old, the incidence is 3% and increases dramatically to 50% among those 85 years or older (Castellani et al., 2010); therefore, the age distribution differences between developed and developing countries might be an influential factor.

Third, some researchers speculate that milder dementia or AD have not been detected due to lack of awareness; older people are highly supported in some countries and reluctant to report their possible mental defects, which all contribute to difficulties in obtaining accurate numbers.

Fourth, epidemiological database is not fully accessible, therefore the prevalence data used to do correlation analysis is incomprehensive, which may bias the accuracy of the correlation statistical analysis. To further demonstrate the correlation between Constipation and Alzheimer’s disease, more extensive data retrieval and analysis were needed.

## Author Contributions

TZ is the main author and wrote the majority of the manuscript. ZS is the corresponding author and substantially contributed to the design of the work. YH and JW participated in interpretation of data for the work. YD, DH, and HD corrected the grammar and vocabulary, and also took part in the data analysis part. All the authors were involved in revising the paper critically for important intellectual content, and agreed to be accountable for all aspects of the work in ensuring that questions related to the accuracy or integrity of any part of the work are appropriately investigated and resolved.

## Conflict of Interest Statement

The authors declare that the research was conducted in the absence of any commercial or financial relationships that could be construed as a potential conflict of interest.
